# Blockley Hospital—Service of W. W. Gerhard, M. D.

**Published:** 1842-07-09

**Authors:** M. W. Wilson

**Affiliations:** Resident Physician


### CLINICAL REPORTS.

### *Blockley Hospital—Service of W. W. Gerhard, M. D.*

Reported by M. W. Wilson, M. D., Resident Physician.

### *Neuralgia.*

Seven cases were treated, three men and four women.

*Neuralgic Cephalalgia successfully treated with Veratria.*—L. S., a man of robust frame, aged 50, entered the ward with severe neuralgia, which had annoyed him for many months. The pain was very severe, and apparently confined to the anterior part of the scalp. After using a variety of means without benefit, he was put on the use of veratria—the twelfth of a grain three times a day for four days, at which time he complained of pain in the stomach, but was entirely relieved from the pain in the head. The medicine was now discontinued, and fifteen grains of the precipitated carbonate of iron given three times a day. He had no return of it while he remained in the ward.

*Case of Neuralgia with some obscure Organic Disease.*—A. T., an English woman, of middle age, and of stout robust frame, had been troubled with various anomalous symptoms for many months. She complained of pain in the head, stomach and breast, and occasionally in her back between the shoulders. At most times her countenance exhibited the appearance of anemia, but sometimes it was flushed, and her expression was that of suffering.

The extremities occasionally became swollen and œdematous; and her urine, by the application of the tests—heat and nitric acid—deposited albumen. The œdema and albuminous urine, which no doubt depended on functional derangement rather than organic disease of the kidneys, entirely left her. The pulse was bounding, full and frequent, ranging from 80 to 120 per minute; and although reduced by a full bleeding, it returned to its former standard within twenty-four hours.

The physical signs indicated no disease of the chest, except slight hypertrophy of the heart. The anemia, the occasional dropsy, and the peculiar character of pulse, were symptoms not easily explicable, except from the supposition of some organic disease; and by way of exclusion, a doubtful but probable diagnosis was arrived at, namely aortitis.

[This case was a most obscure one. The patient had been a woman originally in easy circumstances. She fell into great poverty, and afterwards became intemperate. The most prominent symptoms were dyspnœa and frequency of the pulse; the neuralgia was also severe, but much more variable. The anemic state of the patient had formed after many of the other symptoms were developed, and could not at any rate have done more than increase the neuralgic pains. Knowing that chronic aortitis is often connected with some of these symptoms, (the dyspnœa and excited pulse,) and finding no other obvious lesion, we referred the disorder to this source as its possible, perhaps probable cause.—W. W. G.]

*Spinal Irritation.*—We had the usual signs of pain in the stomach and breast, which was very much increased by pressure on the spine, in points corresponding with the origin of the nerves, supplying the places where the pain was seated. The disease yielded readily to the application of cups and frictions of croton oil along the spine.

The remaining cases of this class of diseases—neuralgia—deserve no particular notice, as there were no points of interest connected with them.

### *Paralysis.*

Three cases were treated.

Case I. *Paralysis from Dysentery.*—H. H., ætat. 40, entered the ward with paralysis of the lower extremities. From the intelligence we could get from the patient, (who was intoxicated when he entered the ward, and whose memory was rather feeble at all times,) it supervened on an attack of acute dysentery with hæmorrhage from the bowels. The hæmorrhage occurred about four weeks previous to his entrance, and was immediately followed by the loss of power of his lower extremities. When he came under our notice he was capable of moving his legs with facility when lying in bed, but he was unable to support his weight. The active inflammatory symptoms of dysentery had passed off, but his bowels were open very frequently. He was treated with small doses of the pulv. ipecac. et opii., and mucilaginous diet. He improved very fast, and was able to support his weight, and walk across the ward within a week. He was discharged well, with the exception of an awkward walk that usually attends convalescence from paralysis.

Case II. *Paralysis from Inflammation of the Spinal Cord.*—E. C. ætat. 57, has had several attacks of paralysis previously, which readily yielded to antiphlogistic treatment, with counter-irritants along the spine. He entered the ward now for the fourth time similarly affected. He complains of pain in the back and between the shoulders. The paralysis was imperfect in both the upper and lower extremities. He was cupped along the spine, and purged frequently. Under this treatment, which is precisely the same that was used in the previous attacks, he convalesced in about three weeks, but remained in the ward for near eight weeks. He did not become entirely free from the paralysis, but was so well that he could walk and use his limbs with facility.

The other case was a young man convalescent from hemiplegia, which followed a stroke of apoplexy. He was convalescent when he entered the ward.

*Rheumatism.*—Seventeen cases were treated. These cases were, for the most part, chronic. They had been of long standing—from two months to three years or more. They were treated with anodynes, and the iodide of potassium. The more recent cases were very much relieved, or entirely cured, while little effect was produced on those of very long standing.

[The iodide of potash is certainly one of the best remedies we possess in chronic rheumatism ; but like all others it is at times uncertain. In the hospital it is very difficult to judge of the effects of remedies in the chronic varieties of rheumatism without pyrexia, for many of the patients are so habituated to complaint, and so unwilling to admit their recovery, that it is often difficult to say when they are really much improved. Besides, all remedies in chronic rheumatism relieve it for a time only. Our method of administering the iodide of potass, is to give from five to ten grains three times daily, with an opiate at night.—W. W. G.]

*Intermittent Fever.*—Four cases came under our notice. This disease assumed a very mild form this season. It was checked in every case by the administration of ten grains of the sulphate of quinine an hour before the anticipated rigour, followed by forty drops of the tincture of opii just as the chill was commencing. In no case did there exist a necessity for a second dose.

[The practice of the hospital has always been to give the quinine in comparatively large doses. Of late years this method has become more general: and the dose is now rarely less than five grains.]

